# Therapeutic Efficacy of Carvacrol-Loaded Nanoemulsion in a Mouse Model of Schistosomiasis

**DOI:** 10.3389/fphar.2022.917363

**Published:** 2022-06-17

**Authors:** Edilaine S. Xavier, Rafael L. de Souza, Vinícius C. Rodrigues, Camila O. Melo, Daniel B. Roquini, Bruna L. Lemes, Polrat Wilairatana, Elquio E. Oliveira, Josué de Moraes

**Affiliations:** ^1^ Research Center for Neglected Diseases, Guarulhos University, Guarulhos, Brazil; ^2^ Laboratory of Synthesis and Drug Delivery, State University of Paraiba, João Pessoa, Brazil; ^3^ Department of Clinical Tropical Medicine, Faculty of Tropical Medicine, Mahidol University, Bangkok, Thailand

**Keywords:** nanotechnology, antischistosomal compounds, drug delivery, nanoemulsion, natural product, infectious diseases

## Abstract

Since praziquantel is the only drug available to treat schistosomiasis, a neglected parasitic disease that affects more than 240 million people worldwide, there is an urgent demand for new antischistosomal agents. Natural compound-loaded nanoparticles have recently emerged as a promising alternative for the treatment of schistosomiasis. Carvacrol is an antimicrobial monoterpene present in the essential oil extracted from several plants, especially oregano (*Origanum vulgare*). In this study, a carvacrol nanoemulsion (CVNE) was prepared, characterized, and administered orally (200 mg/kg) in a mouse infected with either immature (prepatent infection) or adult (patent infection) *Schistosoma mansoni*. For comparison, data obtained with an unloaded nanoemulsion (blank formulation), free carvacrol, and the drug of reference praziquantel are also presented. CVNE was more effective than free carvacrol in reducing the worm burden and egg production in both patent and prepatent infections. Favorably, CVNE had a high effect in terms of reducing the number of worms and eggs (85%–90%) compared with praziquantel (∼30%) in prepatent infection. In tandem, carvacrol-loaded nanoemulsion markedly improved antischistosomal activity, showing efficiency in reducing worm and egg burden, and thus it may be a promising delivery system for the treatment of schistosomiasis.

## Introduction

Schistosomiasis, caused by infection with an intravascular flatworm (blood flukes) of the genus *Schistosoma*, remains one of the most prevalent parasitic diseases. Endemic in 78 countries, schistosomiasis affects more than 250 million people and approximately 10% of the world’s population is at risk of infection (World Health Organization, 2022). Considered a neglected disease, schistosomiasis disproportionally affects the poorest and most deprived communities, causing substantial morbidity and even mortality. Morbidity due to schistosomiasis results from immunologic reactions to *Schistosoma* eggs trapped in tissues, causing various complications from inflammation and granulomatous reactions (McManus et al., 2018). In children, the disease can also be devastating for growth, resulting in stunted physical and cognitive development (Mduluza and Mutapi, 2017).

Schistosomiasis treatment and control depend almost entirely on a single drug, praziquantel (Lago et al., 2018). This drug is listed in the WHO Model List of Essential Medicines, and it has been used on a large scale for decades (World Health Organization, 2021a). Although effective against all Schistosome species, praziquantel displays low efficacy against immature parasite stages (prepatent infection), which represents a major gap in terms of therapeutic intervention (Spangenberg, 2021). In addition, low cure rates and the reduced efficacy of praziquantel after multiple rounds of mass drug administration have recently been reported (Zwang and Olliaro, 2017; Kabuyaya et al., 2018). In addition, praziquantel is available in tablet form, and a high dose (40–60 mg/kg) is required, making the treatment of young children extremely difficult. Given the critical role these conditions play in global health, the World Health Organization launched in 2021 a new roadmap for neglected diseases for 2021–2030, with strategies to accelerate the control and elimination of schistosomiasis and other poverty-associated diseases by 2030 (World Health Organization, 2021b). As highlighted in the WHO roadmap, the development of novel therapeutic interventions is needed to eliminate schistosomiasis as a public health problem by 2030. Among the strategies to improve drug performance for the treatment of schistosomiasis, the use of nano-based drug delivery systems has been cited as an interesting approach (Mengarda et al., 2022).

In recent years, many natural compounds have attracted attention for their interesting antischistosomal activities (Mafud et al., 2018; Mengarda et al., 2020; Sessa et al., 2020; Mengarda et al., 2021). In addition, natural compound-loaded nanoparticles have recently emerged as a promising alternative for the treatment of schistosomiasis. In this context, several research groups have published nanotechnological solutions to improve the efficacy of poorly soluble compounds administered orally (De Lima et al., 2018; Silva et al., 2021a). Carvacrol is an antimicrobial monoterpene that is widely used as a flavoring agent in food and cosmetics. It is present in the essential oil extracted from several plants, especially oregano (*Origanum vulgare*). Carvacrol’s antimicrobial properties are higher than those of other volatile compounds present in essential oils due to hydrophobicity, the presence of the free hydroxyl group, and the phenol moiety (Sharifi-Rad et al., 2018). Given the chemical and physical characteristics of carvacrol, the use of an appropriate nano-based drug delivery system is becoming increasingly widespread (He et al., 2019; Niza et al., 2020; Miranda-Cadena et al., 2021; Vitali et al., 2021). Previous studies have described a stable carvacrol-loaded nanoemulsion with absence of cytotoxicity (Dantas et al., 2021; Motta Felício et al., 2021). Moreover, carvacrol nanoemulsion exhibited improved antimicrobial activity compared to the free oil (Motta Felício et al., 2021).

In this study, a carvacrol-loaded nanoemulsion (CVNE) was produced and characterized. Next, the antiparasitic properties of CVNE were determined *in vivo* using either an early or a chronic *Schistosoma mansoni* murine model to characterize the full spectrum of activity. For comparison, data obtained with an unloaded nanoemulsion (blank formulation, BNE), free carvacrol, and the drug of reference praziquantel are also presented.

## Materials and Methods

### Materials

Carvacrol oil (5-isopropryl-2-methylphenol, purity 98%), sorbitan monostearate 80 (Span 80^®^), and Tiazolyl blue tetrazolium bromide (MTT) were purchased from Sigma–Aldrich (St. Louis, MO, United States). Polysorbate 80 (Tween 80^®^) was provided by Vetec Química Fina LTDA (Duque de Caxias, RJ, Brazil). Medium chain triglyceride (Mygliol 812^®^) was acquired from Sasol (Brunsbüttel, Germany). RPMI 1,640 culture medium Dulbecco’s Modifed Eagle Medium (DMEM), inactivated fetal bovine serum and antibiotics (10,000 units/ml Penicillin G sodium salt and 10 mg/ml streptomycin sulfate) were purchased from Vitrocell (Campinas, SP, Brazil). Praziquantel was kindly provided by Ecovet Indústria Veterinária Ltda (São Paulo, Brazil).

### Preparation and Characterization of Carvacrol Nanoemulsion

Carvacrol nanoemulsion (CVNE) was prepared by ultrasonic emulsification as previously described (Motta Felício et al., 2021). The nanoemulsion was formulated with 3% of carvacrol oil, 9% of surfactants (mix of Tween 80 and Span 80) and 88% of water using an ultrasonic probe apparatus (model QR200, Ultronique, Indaiatuba, SP, Brazil) at 300 W. The blank nanoemulsion (BNE) was formulated replacing carvacrol by Mygliol 812^®^. Globule size, polydispersity index (PDI) and zeta potential of nanoemulsion were analyzed using the Zetasizer equipment (Nano ZS, Malvern Instruments, United Kingdom) using dynamic light scattering, performed at a scattering angle of 173° at 25°C. The pH of the CVNE was measured using a multipurpose autotitrator (model MPA-210, MS Tecnopon Instruments, Piracicaba, SP, Brazil), previously calibrated with buffer solutions of pH 4.0 and 7.0. The analyses were performed after 1, 15, 60, and 90 days of formulation. The nanoemulsions were stored in 10 ml glass vials sealed with plastic caps and kept refrigerated at 4 ± 2°C.

The carvacrol content in CVNE was measured by ultraviolet-visible spectrophotometry (model Genesys 10S UV-Vis, Themo Fisher Scientific, Germany) at 276 nm, using a calibration curve (*n* = 6; y = 0.0158x—0.0265; *R* ^2^ = 0.9965). The formulation was diluted in ethanol in the proportion of 1:2000 and filtered using 0.2 µm membrane filter before analysis.

### Animals and Parasite

The life cycle of *S. mansoni* (BH strain) is maintained by passage through *Biomphalaria glabrata* snails (intermediate host) and Swiss mice (definitive host) at Guarulhos University (São Paulo, Brazil). Female mice (age 3 weeks; weight ca. 20–22 g) were purchased from Anilab (São Paulo, Brazil). Both snails and mice were kept under environmentally controlled conditions (temperature ∼25°C; humidity ∼70%; 12 h light- and 12 h dark cycle) with free access to water and food. For parasite maintenance, rodents were infected with *S. mansoni* by subcutaneous injection of approximately 120 cercariae previously harvested from infected snails by exposure to light for 3 h, according to standard procedures from our laboratory (Lago et al., 2019; Porto et al., 2021).

### *In Vivo* Drug Treatments

Mice were infected as described above by subcutaneously injecting approximately 80 *S. mansoni* cercariae, and *in vivo* studies was performed as previously described (Guerra et al., 2019; Roquini et al., 2019). Animals were then randomly divided into experimental groups (five mice per group) and CVNE was tested using a single oral dose (200 mg/kg) 21 days post-infection (immature stage and prepatent infection) or 42 days post-infection (adult stage and patent infection). For comparison, groups of *S. mansoni*-infected control mice were given a corresponding amount of BNE or Phosphate Buffered Saline (PBS) on the same timetable. Praziquantel at 400 mg/kg (therapeutic dose for experimental schistosomiasis in a mouse model) was also administered to *S. mansoni*-infected rodents in the same period. On day 63 post-infection, animals in all groups were weighed and euthanized following standard and international protocol (Silva et al., 2017; Silva et al., 2021b). For determination of worm burden, schistosomes were collected by portal perfusion (Lombardo et al., 2019; Silva et al., 2021c). Therapeutic efficacy was also based on the Kato-Katz method for quantitative examination of fecal eggs, and the number of eggs per gram was calculated (De Brito et al., 2020; Xavier et al., 2020). The percentage of worm and egg reduction was calculated by means of the following equation: % reduction = [(value of untreated control group—value of treatment group)/value of untreated control group] × 100% (Mengarda et al., 2021). Analyses were conducted by two different investigators according to standard procedures (Amorim et al., 2020; Porto et al., 2021).

### *In vitro* Antiparasitic Assay

*In vitro* antischistosomal assay was performed as previously described (Sessa et al., 2020; Mengarda et al., 2021). Briefly, adult *S. mansoni* worms were recovered by perfusion from mice at day 42 post infection. Next, parasites were placed in RPMI 1640 medium supplemented with 5% (vol/vol) fetal bovine serum, containing 100 μg/ml streptomycin and 100 IU/ml penicillin and incubated in a 24-well culture plate (Corning, New York, NY, United States) containing two pair of parasites per well. CVNE, BNE and free carvacrol were tested using 1:2 serial dilutions from 12.5 to 200 μM for determination of their 50% effective concentration (EC_50_). Each concentration was tested in triplicate, and experiments were repeated once. Parasites were kept for 72 h (37°C and 5% CO2), and their viability was monitored microscopically.

### Cytotoxicity Assay

The CVNE and BNE were evaluated for their cytotoxicity against HaCaT (human epithelial cells) and Vero (monkey kidney cells) using the MTT assay as previously described (Morais et al., 2021). Briefly, cells were plated at 2 × 10^3^/well in 96-well culture plates in DMEM enriched with 10% fetal bovine serum and incubated overnight at 37°C and 5% CO_2_. Next, nanoemulsions were added in 3-fold serial dilutions (starting at 200 μM), each concentration in triplicate, and the plates were incubated for 72 h at 37°C and 5% CO_2_. Next, 20 µl of MTT was added to each well, and the plates were incubated for an additional 4 h. The absorbance was measured at 595 nm using a spectrophotometer (Epoch Microplate Spectrophotometer, BioTek Instruments, Winooski, VT, United States). Cytotoxicity was evaluated in two independent assays.

### Statistical Analysis

All statistical analyses were performed using Graph Pad Prism software 8.0 (Graphpad software Inc., CA, United Ststes). The difference was considered statistically significant if *P* < 0.05 using the non-parametric Kruskal Wallis test. The data and statistical analysis comply with the recommendations on experimental design and analysis in pharmacology (Lago et al., 2019).

## Results

### Preparation and Characterization of Oil-In-Water Nanoemulsions

**FIGURE 1:**
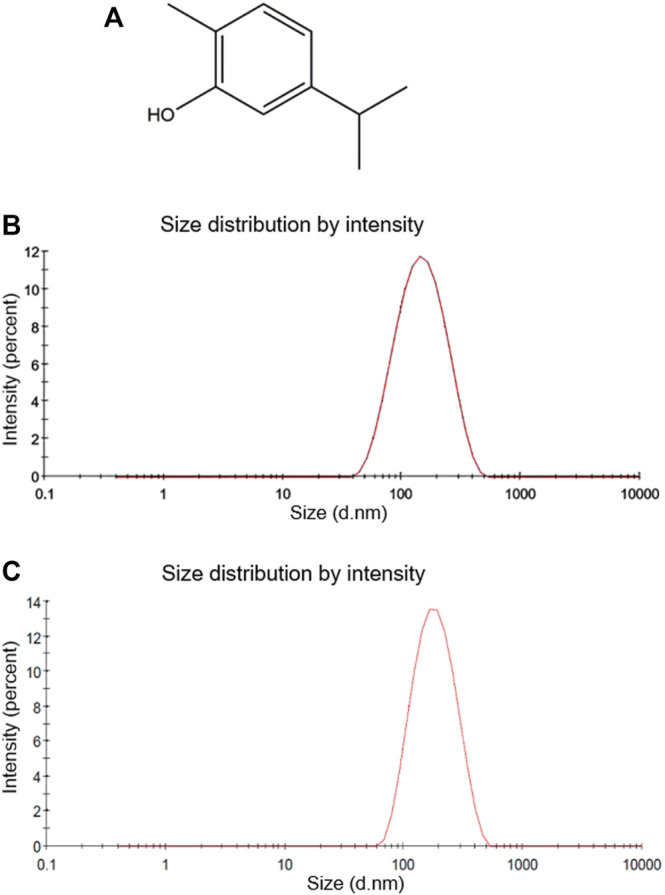
Chemical structure and droplet size distributions. **(A)** Chemical structure of carvacrol. **(B)** Carvacrol nanoemulsion droplet size distribution curve by intensity after 1 day of formulation **(C)** Carvacrol nanoemulsion droplet size distribution curve by intensity after 90 days of formulation.

Using the monoterpene carvacrol (Figure 1A), the CVNE was developed by the ultra-sonic method. As shown in Table 1 and in the size distribution curve (Figures 1B,C), CVNE exhibited a monodispersed characteristic, with narrow distribution, where the nanoemulsion had an average droplet diameter of 124 and 165 nm after one and 90 days of preparation, respectively. The ultrasonic method was chosen for the CVNE formulation because it can produce small droplets due to turbulence and cavitation formed in the system (McClements, 2011; Nirmala et al., 2020; Wu et al., 2021). In this way, the mixture of non-ionic surfactants present in the formulation could interact with the oil phase and prevent the droplets from re-aggregating.

**TABLE 1:** Mean droplet diameter, polydispersity index (PDI), zeta potential and pH of carvacrol nanoemulsion (CVNE), and blank nanoemulsion (BNE) over 90 days.

Parameter of stability/Day of analysis	Mean droplet diameter (nm)	Polydispersity index (PDI)	Zeta potencial (mV)	pH
Day 1	124 ± 0.80	0.20 ± 0.01	−26.4 ± 0.59	5.4 ± 0.04
Day 15	141 ± 0.87	0.17 ± 0.11	−17.4 ± 0.55	5.3 ± 0.04
Day 60	170 ± 2.68	0.16 ± 0.03	−17.4 ± 0.17	5.4 ± 0.03
Day 90	165 ± 0.70	0.12 ± 0.30	−14.3 ± 0.43	5.5 ± 0.01

The PDI values of CVNE were smaller than 0.3. This parameter assesses the degree of uniformity of droplet size distribution within the system (Danaei et al., 2018). PDI values closer to 0 indicate a mono-disperse distribution (Mohd Narawi et al., 2020; Deghiedy et al., 2021). Zeta potential determines the surface charge at the droplet interface, which is indicative of the degree of repulsion between the droplets of the nanoemulsion. Negative charges can increase the rate of repulsion between droplets, preventing destabilization processes such as Ostwald ripening. The CVNE zeta potential varied between −14 and −26, which was indicative of electrostatically stabilized nanoemulsion. The CVNE presented a drug content of 29.84 ± 0.10 mg of carvacrol per mL of nanoemulsion.

### *In Vivo* Studies in an Animal Model of Schistosomiasis

Twenty-one days (prepatent infection) or 42 days (patent infection) post-infection, animals were treated orally with a single dose of 200 mg/kg CVNE. Worm burden and egg production were measured for the CVNE group, and results were compared to the control, namely the infected but untreated rodents that were harboring either prepatent or patent infection. In addition, BNE, free carvacrol, and the antischistosomal drug praziquantel were used for comparison.

### Efficacy of Carvacrol-Loaded Nanoemulsion in Mice Harboring Prepatent *S. mansoni* Infections

**FIGURE 2:**
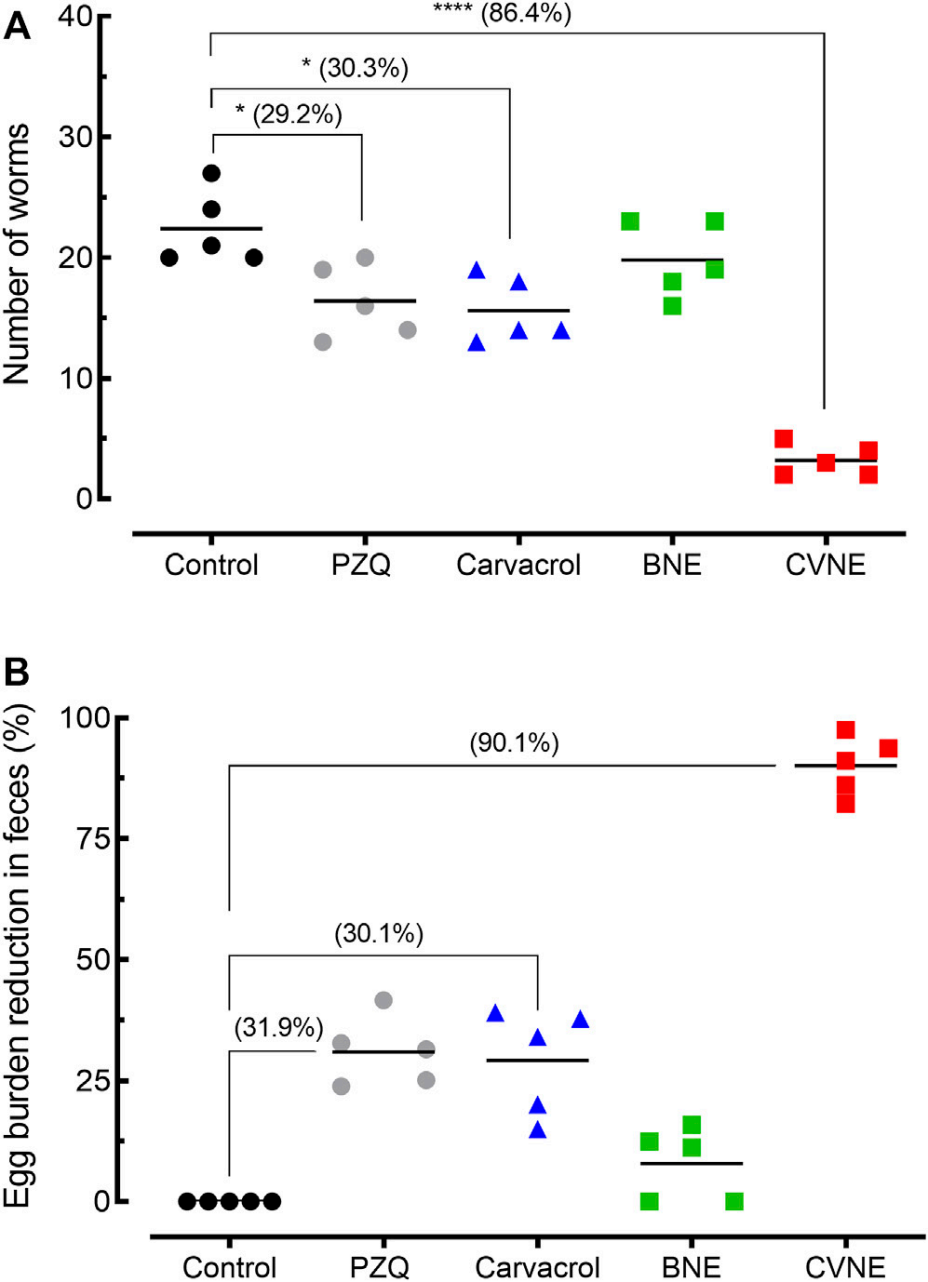
Efficacy of carvacrol nanoemulsion (CVNE), blank nanoemulsion (BNE), free carvacrol, and praziquantel (PZQ) in mice harboring prepatent *Schistosoma mansoni* infection. **(A)** Effect of compounds on worm burden. **(B)** Effect of compounds on egg burden. Drugs were administered orally using a single dose of 200 mg/kg (CVNE and free carvacol) or 400 mg/kg (PZQ) 21 days post-infection to mice harboring juvenile *S. mansoni*. Groups of *S. mansoni*-infected control were given a corresponding amount of vehicle on the same timetable. Points represent data from individual mice (*n* = 5 per group). The percentages of reduction in worms and egg burden are in parentheses. Horizontal bars represent median values. *p < 0.05 and ****p < 0.0001 compared with infected untreated control.

Results of worm burden and egg production in animals harboring prepatent *S. mansoni* infections are summarized in Figure 2. Compared to control *S. mansoni*-infected mice, CVNE dramatically decreased (by 86.4% and *p* < 0.0001) the total number of worms. In contrast, BNE achieved a non-significant worm burden reduction. For comparison, the free carvacrol and the known anthelminthic praziquantel resulted in a low worm burden reduction of 30.3 and 29.2% (*p* < 0.05), respectively (Figure 2A).

With respect to egg burden, a single dose of CVNE was able to reduce the egg burden by 90.1%, whereas administration of BNE did not cause a reduction in the number of eggs recovered in feces when compared to control infected animals. Administration of praziquantel led to a low reduction in the fecal egg burden (31.9%), whereas free carvacrol had similar effect on the egg burden (∼30%) (Figure 2B).

### Efficacy of Carvacrol-Loaded Nanoemulsion in Mice Harboring Patent *S. mansoni* Infections

**FIGURE 3:**
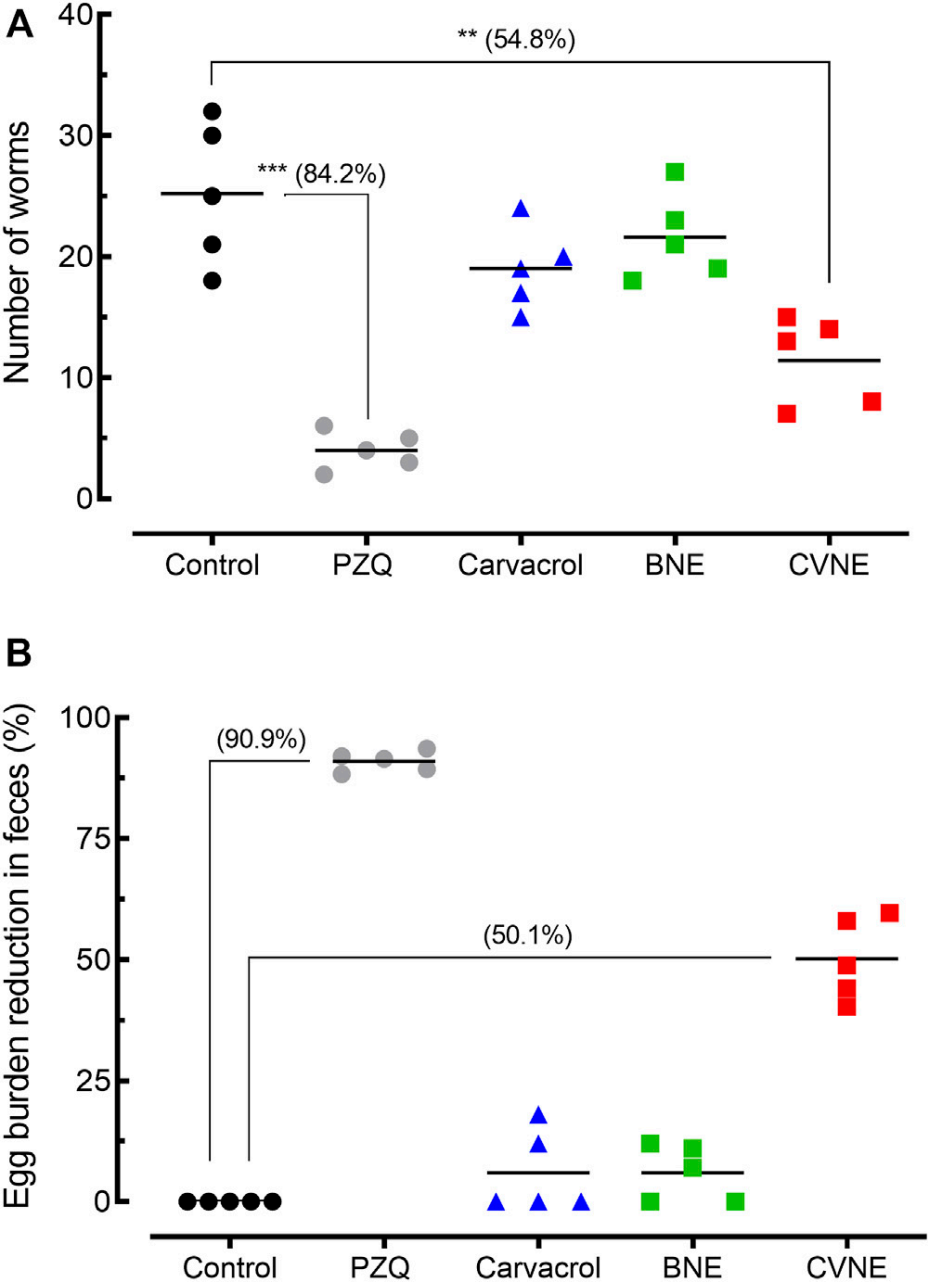
Efficacy of carvacrol nanoemulsion (CVNE), blank nanoemulsion (BNE), free carvacrol, and praziquantel (PZQ) in mice harboring patent *Schistosoma mansoni* infection. **(A)** Effect of compounds on worm burden. **(B)** Effect of compounds on egg burden. Drugs were administered orally using a single dose of 200 mg/kg (CVNE and free carvacrol) or 400 mg/kg (PZQ) 42 days post-infection to mice harboring adult *S. mansoni*. Groups of *S. mansoni*-infected control were given a corresponding amount of vehicle on the same timetable. Points represent data from individual mice (*n* = 5 per group). The percentages of reduction in worms and egg burden are in parentheses. Horizontal bars represent median values. *p < 0.05, ****p < 0.0001 compared with infected untreated control.

Results of worm burden and egg production in animals harboring patent *S. mansoni* infections are summarized in Figure 3. Compared to control *S. mansoni*-infected mice, CVNE achieved a moderate worm burden reduction of 54.8% (*p* < 0.01). The groups of animals treated with BNE or free carvacrol exhibited a non-significant reduction in the number of worms when compared to control infected animals. In contrast, a single dose of praziquantel resulted in a high worm burden reduction of 84.2% (*p* < 0.001) (Figure 3A).

Regarding the egg burden, oral administration of CVNE led to a moderate reduction in the number of eggs in feces (50.1%), whereas BNE and free carvacrol showed a non-significant reduction in the egg production when compared to control infected animal. Higher egg burden reduction values of 90.9% were obtained for praziquantel (Figure 3B).

### *In vitro* Antischistosomal Properties of Carvacrol-Loaded Nanoemulsion

The *in vitro* antischistosomal effect of CVNE and free carvacrol on adult *S. mansoni* was evaluated at different concentrations (6.25–200 μM). In summary, EC_50_ values of 62.37 μM (55.32–71.48 μM) and 56.81 μM (36.79–62.12 μM) were calculated for CVNE and free carvacrol, respectively. BNE had no toxic effect on *S. mansoni* using the same volume of CVNE.

### Cytotoxicity Evaluation

The *in vitro* cytotoxicity effect of CVNE and BNE on HaCat and Vero cells was evaluated at different concentrations (6.25–200 μM). CVNE and BNE showed an average cell viability greater than 95% at all tested concentrations.

## Discussion

Despite the public health importance of schistosomiasis, the current drug-discovery pipeline for this disease is alarmingly unproductive due to the low commercial investments for a disease caused by helminths (Lago et al., 2018). Consequently, as highlighted in the WHO roadmap, there is a strong need to identify novel antischistosomal agents and/or strategies to improve drugs’ performance. Recently, the use of drug nanocarriers has been cited as an interesting approach to improve the efficacy of compounds administered orally (Mengarda et al., 2022). Nanoemulsions are colloidal dispersion forms that can potentially improve the bioavailability and efficacy of many active agents (Håkansson, 2019; Xu et al., 2020). Interestingly, nanoemulsions containing antischistosomal agents have been recently used experimentally for the treatment of schistosomiasis, and these nanocarriers improved the antischistosomal properties (Eissa et al., 2015; Amara et al., 2018; Mengarda et al., 2022). In this study, we prepared and characterized a carvacrol nanoemulsion. Next, we demonstrated that CVNE had significant antiparasitic properties in mice infected with *S. mansoni* when given at both patent and prepatent infections. CVNE was markedly more effective than free carvacrol in terms of worm and egg burden reduction, demonstrating the possibility of the nanoemulsion improving carvacrol activity.

The carvacrol nanoemulsion was successfully developed by the ultrasonic method, and the stability of the nanoemulsion was observed over 90 days, exhibiting no visible evidence of creaming or phase separation, in accordance with a previous study (Motta Felício et al., 2021). Zeta potential is an important parameter for the stability of colloidal dispersions and it was assessed to clarify the overall surface charge (Silva et al., 2021a; Motta Felício et al., 2021). The negative zeta potential values could be correlated with the pH and ionic strength of the aqueous phase and this charge is an indication of possible colloidal stability (Müller et al., 2000; Silva et al., 2021a). In addition to the zeta potential and PDI, it is known that the size of nanoparticles is an important property for antischistosomal activity. Indeed, among nanoparticles from 55 to 500 nm, smaller particles produced better antischistosomal results (De Souza et al., 2014; Eissa et al., 2015). Moreover, nanoparticles with average size below 400 nm were able to cross the intestinal cell barrier (Doktorovová et al., 2016). In this regard, the average size obtained for the CVNE in this study (120 nm) was satisfactory for carrying out studies in animals.

A single dose of 200 mg/kg of CVNE given to mice infected with either juvenile stage (prepatent infection) or adult stage (prepatent infection) of *S. mansoni* resulted in a high worm burden reduction when compared to free carvacrol. Egg production is contingent on worm maturation and pairing of adult females and males. Females lay hundreds of eggs per day, and many of these eggs pass through the intestinal wall and are discharged in the feces. Treatment of *S. mansoni*-infected mice with CVNE clearly reduced the number of eggs in feces when compared to standard carvacrol. This result could be attributed to a decrease in the number of schistosomes, demonstrating the importance of nanoemulsion for the improvement of antiparasitic activity. Additionally, no anthelmintic activities from the nanoformulation vehicle (BNE) were observed in both *in vitro* and *in vivo* studies, reinforcing the hypothesis that the experimental antiparasitic properties of CVNE are related to the delivery of carvacrol in nanoemulsion. Interestingly, a low cytotoxic potential was observed for CVNE and BNE on two cell lines (HaCat and Vero), which is in accordance to recent observations on peripheral blood mononuclear cells (Dantas et al., 2021).

Comparatively, juvenile parasites appeared more sensitive to CVNE than adult stages, since a reduction of 86.6 and 54.7% in total worm burden was observed in prepatent and patent infection, respectively. Interestingly, in early infection, treatment with CVNE is more effective than the reference drug praziquantel, an important result considering praziquantel’s known lack of activity against the juvenile stage reported in the literature (Sabah et al., 1986; Bergquist and Elmorshedy, 2018). Currently, there is a need for novel drugs that act against juvenile schistosomes, and antischistosomal activity superior to praziquantel in early infection has recently been reported with other antiparasitic agents (De Brito et al., 2020; Xavier et al., 2020; Silva et al., 2021b; Ibrahim et al., 2022). Comparing with studies documented in the literature, CVNE is more effective in mice harboring early *S. mansoni* infection than several other tested compounds (Guerra et al., 2019; De Brito et al., 2020; Xavier et al., 2020; Pereira et al., 2021).

In recent years, several studies have demonstrated that infections by schistosomes are associated with alterations of the gut microbial profiles (Jenkins et al., 2018; Cortés et al., 2020; Zhang et al., 2020). Theses findings suggest that the baseline composition of the host gut microbiome might impact host susceptibility to schistosomes colonization, as well as infection-associated changes in gut microbial profiles. In addition, the depletion of the gut microbiota by antibiotics attenuates granuloma formation of *Schistosoma*-infected mice (Zhang et al., 2020). Since the antimicrobial properties of CVNE has been described (Motta Felício et al., 2021), it may be possible that the worm and egg burden in mice harboring either prepatent or patent *S. mansoni* infection are also influenced by the reduction of the gut microbiota. However, further studies (e.g., histopathology of liver and evaluation of immune response), are needed to verify the influence of CNVE in the pathology of schistosomiasis.

The exact mechanism by which carvacrol exerts its antiparasitic action on schistosomes is still not clear. However, like other terpenes, carvacrol has high hydrophobicity, allowing it to penetrate across membranes, and to interact with intracellular proteins and/or intra-organelle sites. It is known that carvacrol inactivates micro-organisms through a multi-target action that disrupts cell membranes and inhibits the respiratory activity (Swamy et al., 2016). The antischistosomal activities of several terpenes have been reported (Mafud et al., 2016; Silva et al., 2017; Sessa et al., 2020), but further studies are needed to elucidate their mechanism of action.

Carvacrol is listed as a Generally Recognized as Safe (GRAS) food additive by the United States Food and Drug Administration (Hallagan and Hall, 1995). The low cost of production of carvacrol and its safety make it attractive as not only as a food additive, but also as a therapeutic agent (Sharifi-Rad et al., 2018). However, carvacrol is slowly absorbed in the intestine after oral administration, with more than 30% remaining in the gastrointestinal tract (Suntres et al., 2015; Sharifi-Rad et al., 2018), which may explain its low activity against blood parasites such as *S. mansoni*. In this context, carvacrol-loaded nanoemulsion markedly improved antischistosomal activity and, thus, nanoemulsions appear to be a substantiated alternative in the treatment of diseases caused by blood helminth parasites.

## Conclusion

Carvacrol is a promising natural monoterpene that possesses a wide range of biological activities, including antimicrobial properties (Silva et al., 2021a). However, carvacrol is poorly absorbed after oral administration, which may limit its effect against blood worms such as schistosomes. Since nanoemulsions have been successfully used to improve the bioavailability of many active agents, in this study carvacrol nanoemulsion (CVNE) was prepared, characterized, and administered orally in mice infected with either immature (prepatent infection) or adult (patent infection) *S. mansoni*. The obtained CVNE presented a droplet size smaller than 200 nm, satisfactory Zeta potential, homogeneous distribution, and good stability. Comparatively, a single oral 200 mg/kg dose of CVNE significantly reduced worm burden and egg production in both prepatent and patent *S. mansoni* infection when compared to free carvacrol, clearly demonstrating that the nanoformulation improved drug efficacy. In patent infection, CVNE yielded moderate efficacy compared with the drug of reference praziquantel. On the other hand, CVNE was more effective than praziquantel in prepatent infection. Furthermore, CVNE showed no cytotoxicity at concentrations up to 200 µM in HaCat and Vero cells. These findings represent a good starting point for initiatives regarding antischistosomal drug discovery using nanotechnological solutions to improve the efficacy of antiparasitic compounds administered orally.

## Data Availability

The raw data supporting the conclusion of this article will be made available by the authors, without undue reservation.
